# Assessment and Treatment of Abuse Risk in Opioid Prescribing for Chronic Pain

**DOI:** 10.1155/2011/941808

**Published:** 2011-10-11

**Authors:** Robert N. Jamison, Juliana Serraillier, Edward Michna

**Affiliations:** Pain Management Center, Department of Anesthesiology, Perioperative and Pain Medicine, Brigham and Women's Hospital, Harvard Medical School, 850 Boylston Street, Chestnut Hill, MA 02467, USA

## Abstract

Opioid analgesics provide effective treatment for noncancer pain, but many physicians have concerns about adverse effects, tolerance, and addiction. Misuse of opioids is prominent in patients with chronic back pain and early recognition of misuse risk could help physicians offer adequate patient care while implementing appropriate levels of monitoring to reduce aberrant drug-related behaviors. In this review, we discuss opioid abuse and misuse issues that often arise in the treatment of patients with chronic back pain and present an overview of assessment and treatment strategies that can be effective in improving compliance with the use of prescription opioids for pain. Many persons with chronic back pain have significant medical, psychiatric and substance use comorbidities that affect treatment decisions and a comprehensive evaluation that includes a detailed history, physical, and mental health evaluation is essential. Although there is no “gold standard” for opioid misuse risk assessment, several validated measures have been shown to be useful. Controlled substance agreements, regular urine drug screens, and interventions such as motivational counseling have been shown to help improve patient compliance with opioids and to minimize aberrant drug-related behavior. Finally, we discuss the future of abuse-deterrent opioids and other potential strategies for back pain management.

## 1. Introduction

Chronic back pain negatively impacts every facet of daily living. Chronic back pain, defined as pain that lasts longer than 6 months, has been seen to interfere with individual's quality of life, by interrupting sleep, employment, social functioning, and many other daily activities. Patients with chronic back pain typically report feelings of depression, anxiety, irritability, sexual dysfunction, and decreased energy. Often chronic back pain adversely affects family roles and contributes to worry about financial limitations and future disability [1–5].

Studies analyzing factors affecting health and illness have shown that chronic pain, of which chronic back pain is the most prominent, is a widespread international problem [6–8]. More than 90 million Americans show symptoms of chronic pain, which is approximately one third of the US population. In the United States, chronic pain accounts for 21% of emergency department visits and 25% of annual missed workdays. Chronic pain is responsible for up to $100 billion in annual direct and indirect costs, making it the most financially challenging condition to date [9–12]. 

Opioid analgesics have been used to help manage acute as well as cancer-related pain [13]. This class of prescription medication is also used as a treatment for individuals with chronic noncancer back pain; however, many physicians are reluctant to prescribe opioids for these patients since this medication contributes to adverse effects, tolerance, and addiction [14].

The National Comorbidity Survey of Psychiatric Disorders collected epidemiological data, indicating a lifetime prevalence of 7.5% for drug dependence (illicit or prescription drugs) and 14.1% for alcohol dependence for individuals in the United States [15]. The Diagnostic and Statistical Manual of Mental Disorders (DSM-IV) results showed approximately 3% of US citizens 18 years or older met the criteria for illicit drug abuse or dependence [16]. Another study that used a sample of 363 inpatients between the ages of 18 to 49 found that 21.8% of the participants had a current addiction to alcohol or illicit drugs [17].

There has been a steady increase in the use of opioids in the United States [18]. This has been due to increasing pain awareness, support from pain organizations, changes in treatment guidelines, increasing patient understanding of pain, new formulations, and industry pressure. It is estimated that 235 million opioid prescriptions were written in the United States in 2004 alone [19]. The nonmedical use of prescription opioids has also continually increased among all ages, and now prescription opioid analgesics are reported to be the most frequently abused drugs in the United States [20]. Misuse of opioids is also prominent in patients with chronic back pain, and unintentional drug overdose due to prescription opioids has continued to increase since 1970 (http://www.cdc.gov/HomeandRecreationalSafety/pdf/poision-issue-brief.pdf,  Accessed 4/16/10). A literature review done by Strain [21] reported that 15% to 23% of patients with chronic pain met the criteria for a substance abuse disorder, suggesting that this continues to be a problem. 

The pain literature suggests that physicians are able to better provide suitable treatment and care to patients with chronic pain once substance misuse causes are recognized [22]. Misuse behaviors of prescribed opioid medication are determined by assessment and treatment protocols. These protocols help to identify patients who show signs of opioid misuse, since it provides the clinicians with an overview of the patient's background and behavior.

In this paper, we review opioid abuse and misuse issues that often arise in the treatment of patients with chronic noncancer back pain and discuss assessment and treatment strategies that can be effective in improving compliance with the use of prescription opioids for pain. The intent of this review paper is to provide a brief overview of studies on the assessment and treatment of abuse risk on opioid prescribing for chronic noncancer pain. This paper is not a systematic review, but rather a critical discussion of current assessment techniques and procedures within the clinical setting. The paper is divided into the following subsections: (1) definition of terms, (2) adverse effects of opioid therapy, (3) medical comorbidity among pain patients, (4) initial medical assessment, (5) psychiatric comorbitidity, (6) substance abuse assessment, (7) urine toxicology screens, (8) opioid therapy agreement, (9) abuse-deterrent opioids, (10) interventions for high-risk patients, and (11) future considerations.

## 2. Definition of Terms

A clear definition of terms helps to minimize confusion and to clarify the objectives of therapy with patients taking opioid analgesics for pain (Appendix A). *Substance misuse* is the use of any drug in a manner other than how it is indicated or prescribed, while *substance abuse* is defined as the use of any substance when such use is unlawful or when such use is detrimental to the user or others. *Addiction* is a behavioral pattern of substance abuse characterized by overwhelming involvement with the use of a drug. Addiction is generally understood to be a chronic condition from which recovery is possible; however, the underlying neurobiologic dysfunction, once manifested, is believed to persist [16, 23]. Addiction focuses on compulsive use of the drug that results in physical, psychological, and social harm to the user. An individual who has an addiction to a drug continues to use it, despite harm. *Physical dependence* is a common phenomenon of all mammals taking opioids characterized with physical withdrawal symptoms when an opioid is discontinued. *Tolerance* is also a commonly observed phenomenon when taking opioids over time in which the individual becomes used to the drug and has a need for increasing doses to maintain the same effect. Both physical dependence and tolerance are typically found among patients who use opioids for chronic pain and are unrelated to true addiction. Finally, *aberrant drug-related behavior* is behavior suggestive of a substance abuse and/or addiction disorder. Examples are selling prescription drugs, prescription forgery, stealing or “borrowing” drugs from others, injecting oral formulations, obtaining prescription drugs from nonmedical sources, multiple episodes of prescription “loss,” repeatedly seeking prescriptions from other clinicians, evidence of deterioration in function (work, home, and family), and repeated resistance to change therapy despite evidence of physical and psychological problems.

Several authors have shown that the majority of those taking opioids for the treatment of pain typically do not develop addiction or substance use disorders [24], although most patients on long-term opioid therapy develop physical dependence and tolerance to the medication. Those who are undermedicated may demonstrate drug-seeking behaviors or try and self-manage unauthorized dosage increases in an attempt to find relief. Among many of these patients, once adequate relief from the pain is obtained, the drug-seeking behaviors, otherwise known as pseudoaddiction, disappear [25]. However, the long-term efficacy of opioid therapy has been questioned and is estimated that less than 40% reach a 35% improvement in pain intensity [26].

## 3. Adverse Effects of Opioid Therapy

There are a number of adverse effects associated with chronic opioid therapy including nausea, sedation, and opioid-induced bowel dysfunction. There has been a suspected relationship between opioid therapy and a number of conditions such as endocrine deficiencies [27], dose-related cardiac arrhythmia [28], and disordered breathing possibly contributing to unexplained deaths [29]. Despite concerns regarding the use of long-term opioid treatment for pain, few trials exist assessing their efficacy [30]. One study suggested that opioids and NSAIDs may aid short-term pain relief in patients with low back pain; however, stopping treatment may cause an increase in symptoms [31]. It has been seen that some patients become psychologically dependent after long-term opioid use [32, 33]. Other patients who are chronically maintained on high doses of opioids manifest impaired cognition, problems with psychomotor performance, and opioid-induced hyperalgesia [34]. Studies also suggest that a relationship exists between early misuse of opioids and addiction. This relationship emphasizes the need for early detection of risk, close monitoring, and direct interventions when needed.

## 4. Medical Comorbidity among Pain Patients

Many patients with chronic severe intractable back pain may present with several significant medical comorbidities that can affect the course of treatment. Some of the most common comorbidities include asthma, chronic obstructive pulmonary disease, diabetes mellitus, coronary artery disease, hypertension, ulcers, kidney, bladder, and liver problems, or cancer. When patients are asked to rate their level of pain, comorbid conditions may contribute to this rating.

Some individuals suffering from chronic back pain have a history of unhealthy behaviors including minimal exercise, poor diet, and smoking cigarettes. Over time, they experience weight gain and deconditioning. Many chronic pain patients are on multiple medications prescribed by multiple providers, which include blood thinners, blood pressure and heart disease medications, inhalers, and antidepressants. Several chronic pain patients have allergies and reactions to some medications. They may also have medical devices implanted and wear prostheses. It is essential for clinicians to assess and identify current and past medical conditions to avoid any complications.

## 5. Initial Medical Assessment

It should be required for all patients considered for opioid therapy to undergo an extensive initial evaluation, including a thorough medical history, review of past medical records, a urine toxicology screen, and physical examination. For most patients, a psychological evaluation should be conducted, including completion of screening questionnaires. The comprehensive evaluation process should involve identifying other controlled substance prescribers. Opioid prescriptions should only rarely be prescribed to patients on their first visit, and it is important for patients to be informed of the comprehensive assessment process and the policy not to prescribe opioids until all information is obtained. 

A urine toxicology screen should be obtained at the first visit and compared with the patient's recent medication intake in order to identify any opioid misuse. A diagnosis of the patient's back pain should be documented to provide clinicians with the primary pain site and probable cause of pain. It is essential for all of the patient's providers to communicate with one another in order to properly and effectively manage the patient's care. It is important for clinicians to examine several factors, including a patient's age and gender, to provide the best clinical assessment and treatment for the patient. There is evidence to suggest that younger individuals who are prone to impulsivity have a greater risk for misuse of opioids [30, 35, 36]. One recent study of gender differences and opioid misuse suggested that women are at greater risk for misusing opioids due to emotional issues and affective distress, while men tend to misuse opioids due to legal and problematic behavioral issues [37]. Assessment of levels of emotionality is important and, thus, a psychological assessment would also be valuable.

## 6. Psychiatric Comorbidity

Many chronic intractable back pain patients report feelings of depression, anxiety, irritability and have a history of physical or sexual abuse, or a past history of a mood disorder [38, 39]. Close to fifty percent of patients with chronic pain have a comorbid psychiatric condition, and 35% of patients with chronic back and neck pain have a comorbid depression or anxiety disorder [40–42]. In surveys of chronic pain clinic populations, 50% to 80% of patients with chronic pain had signs of psychopathology, making this the most prevalent comorbidity in these patients [43–46]. Studies suggest that most patients with chronic pain present with some psychiatric symptoms. 

One study conducted by Arkinstall and colleagues found a 50% prevalence of mood disorder in patients who were prescribed opioids, showing this to be a common diagnosis for chronic pain patients [47]. Another study found that physicians are more likely to prescribe opioids for noncancer-related pain on the basis of increased affective distress and pain behavior, rather than the patient's pain severity or objective physical pathology [43]. It has been found that patients who have chronic back pain with psychopathology are more likely to report greater pain intensity, more pain-related disability, and a larger affective component to their pain than those who don't have evidence of psychopathology [48, 49]. 

Patients with chronic pain and psychopathology, especially those with chronic low back pain, typically have poorer pain and disability outcome from treatments [50–53]. In studies of patients with both chronic pain and anxiety and/or depression, there was a significantly worse return to work rate one year after injury compared with those without any psychopathology [54, 55]. Patients who had chronic pain with low psychopathology had a 40% greater reduction in pain with IV morphine than those in a high-psychopathology group [56]. It becomes apparent that patients with a high degree of negative affect benefit less from opioids in an attempt to try and control their pain.

Many patients with substance use disorders also have affective disorders. Attempting to manage a comorbid affective disorder may result in decreased substance abuse behaviors, although they may be at risk of relapse [57–60]. Hasin and colleagues found some patients abusing their pain medication as a way to alleviate their psychiatric symptoms [61]. From this finding and other reviews, there is a strong suggestion that individuals with a mood disorder who self-medicate for negative affect are at increased risk for substance abuse [62]. Since many patients with chronic back or neck pain frequently report mood swings and prominent anxiety and depression symptoms, it remains important to carefully monitor all patients for psychiatric comorbidity. A multidisciplinary assessment with input by a psychiatrist or psychologist would be recommended. In this way, individuals who self-medicate with opioids for mood fluctuations have a greater chance to be identified.

## 7. Substance Abuse Assessment

The US Department of Justice recommended efforts to improve identification of abuse and diversion of controlled substances by health care providers [63]. Physicians continue to struggle with providing the appropriate pain relief for patients, while minimizing the misuse of opioid analgesics [64]. Misuse of pain medications includes selling and diverting prescription drugs, seeking prescriptions from multiple providers, using illicit drugs, snorting or injecting medications, and using drugs in a manner other than the way it was intended. 

There are a variety of assessment measures that can be used to help identify those patients who are prone to misuse their pain medications [36]. Structured interview measures have been published for assessment of alcoholism and drug abuse based on DSM-IV criteria [65], but these measures have not been validated in individuals with chronic pain. Some substance abuse measures, including the CAGE Questionnaire, Michigan Alcoholism Screening Test, and Self-Administered Alcoholism Screening Test, were initially designed for other patient populations [66–68]. Using traditional substance abuse assessment tools may be beneficial for patients with a severe substance abuse disorder; however, these assessments may not be useful for individuals with chronic pain since there is a greater chance of a false positive with these measures. In general, there is a risk that medication abuse using traditional substance abuse measures will be identified based on reports of tolerance and dependence when no abuse exists. Validated measures most appropriate for persons with chronic pain are presented in Appendix B. 

The Screener and Opioid Assessment for Patients with Pain-Revised (SOAPP-R) is a 24-item self-administered screening tool developed and validated for those persons with chronic pain who are being considered for long-term opioid therapy. The SOAPP-R is designed to predict aberrant medication-related behaviors [69, 70]. This questionnaire includes subtle items that encourage the patient to admit to certain factors that are positively correlated with opioid misuse yet outwardly are not perceived to lead to reprisals. Any individual who scores more than an 18 on the SOAPP-R is rated as being at risk for opioid misuse. This screening tool has been found to identify 90% of those who will eventually misuse opioids. It has been cross-validated in over 600 patients across the United States. The reliability and predictive validity of the SOAPP-R, as measured by the area under the curve (AUC), were found to be highly significant (test-retest reliability =  .91; coefficient *α* = .86; AUC  =  .74) and were sufficiently similar to values found with the initial sample. A cut-off score of 18 revealed a sensitivity of  .80 and specificity of  .52. Results of a cross-validation suggest that the psychometric parameters of the SOAPP-R are not based solely on the unique characteristics of the initial validation sample [71]. 

The Current Opioid Misuse Measure (COMM) is a 17-item questionnaire developed and validated for patients who have already been prescribed opioids for chronic pain [72]. The COMM helps to identify those patients who are currently misusing their prescribed opioid medication. The COMM is different from other measures that were created to predict misuse behaviors in patients before being prescribed opioids. Rather, the COMM was created to repeatedly document opioid compliance and improve clinician sense of appropriateness of opioid therapy. The COMM has been determined to be a brief but useful self-report measure of current aberrant drug-related behavior. The reliability and predictive validity in this cross validation, as measured by the area under the curve (AUC), were found to be highly significant (AUC =  .79) and not significantly different from the AUC obtained in the original validation study (AUC =  .81). Reliability (coefficient *α*) was.83, which is comparable to the.86 obtained in the original sample [72]. Results of a cross validation suggest that the psychometric parameters of the COMM are not based solely on unique characteristics of the initial validation sample [73]. Both the SOAPP-R and COMM include subtle items that are correlated with opioid misuse and are items patients are willing to answer honestly.

Other validated measures have also been developed to screen patients with pain for addiction risk potential. The 5-item Opioid Risk Tool (ORT), a brief checklist completed by the clinician, is a validated questionnaire that predicts which patients will display aberrant drug-related behaviors [68, 74]. Scores of 8 or higher suggest high risk for opioid medication abuse. A similar rating tool, the DIRE (standing for diagnosis, intractability, risk, and efficacy), is a clinician-rating scale used to predict suitability for long-term opioid treatment for noncancer pain [75]. Scores higher than 14 on the DIRE suggest a greater suitability of opioid therapy for patients with pain. The Pain Assessment and Documentation Tool is yet another scale completed by the clinician, which provides a detailed documentation of the patient's progress, which also helps to objectively record a patient's care [74, 76]. The Screening Instrument for Substance Abuse Potential (SISAP) is a self-report screening questionnaire for substance abuse potential based mostly on the alcohol literature [77]. Unfortunately, these measures, other than the SOAPP-R and COMM, lack cross validation studies. Also, tools to assess risk of opioid misuse were validated for patients with chronic pain but were not designed specifically for patients with chronic back pain. When using any tools to assess risk of opioid misuse, it is essential to have detailed background information about the patient. 

It is important to note that scores of any clinical assessment tool used to determine abuse risk are not necessarily reason to deny opioids, but rather provide an estimate of the level of appropriate monitoring for the patient. Thus, although these clinical assessments are useful to estimate risk of noncompliant opioid use, the results are most useful to help determine how closely to monitor patients during opioid therapy.

Patients who are typically at a lower risk for misusing opioids include those who are older, generally compliant, have a record of rarely misusing any medication, show stable mood, are thoughtful and responsible, and generally have an easy-going personality. Risk factors for opioid misuse include (1) family or personal history of substance abuse, (2) young age, (3) history of criminal activity and/or legal problems (e.g., charged driving under the influence, DUI), (4) frequent contact with high-risk individuals or environments, (5) history of previous problems with employers, family, and friends, (6) history of risk-taking/thrill-seeking behavior, (7) smoking cigarettes, (8) history of severe depression or anxiety, (9) multiple psychosocial stressors, and (10) previous drug and/or alcohol rehabilitation (see Appendix C). Patients prescribed opioids should be monitored regularly and should be examined for experiencing any adverse effects. Appropriate follow-up care should include repeated psychological evaluations.

## 8. Urine Toxicology Screens

Clinicians use urine drug screens in order to closely monitor patient's adherence to their prescribed opioid medication. Highly sensitive and specific urine screens (e.g., gas chromatography/mass spectrometry—GC/MS) help to identify presence and quantities of prescription medications, presence of illegal substances, and/or absence of prescribed medications. Even though many patients prescribed opioids do not misuse their medication, it still is important to document compliance for all patients on chronic opioid therapy by obtaining a urine screen at least yearly. 

The combination of urine screens, self-report questionnaires, and behavioral observation methods has allowed physicians to properly identify which patients are misusing their prescribed opioids. One study gathered urine toxicology results among 122 patients who were prescribed opioids for noncancer pain and found abnormal results in 43% of this sample [78]. Another study found 21% of the study patients with no obvious behavioral issues to have either a positive urine screen result for an illicit drug or a nonprescribed controlled medication. These results imply that some risk factors for opioid misuse may not always properly identify patients who do misuse their pain medication. An additional study of 226 patients primarily with chronic back pain surprisingly found 46.5% of the sample to have abnormal urine toxicology screen results [35]. In a retrospective study of 470 patients, 4 of 10 patients prescribed opioids also had abnormal urine toxicology screens [79]. These studies underscore the importance of urine toxicology screens along with behavioral observation and self-report measures to help identify aberrant drug-related behavior. Although immunoassay urine screens are often used as the first line of analysis, gas chromatography/mass spectrometry urine screens provide useful results in being able to quantify the extent of illicit and prescription drug use. This type of urine toxicology screen is also able to detect drug metabolites, as well as determine whether the patient has attempted to adulterate the urine sample. 

Increasingly, patients with chronic pain are using marijuana for the treatment of their symptoms, and evidence of THC in the urine has become more prevalent [80]. There is an ongoing debate about the use of medical marijuana and specifically its use among pain patients prescribed opioids for pain [81]. Some clinicians feel that in order to be prescribed opioids, there needs to be a record of clean urines and that use of marijuana is unacceptable when taking prescription opioids because of its association with abuse of other illicit substances. Others feel that use of marijuana is not grounds for discontinuation of opioid therapy, although legal implications need to be considered. Nonetheless, the use of regular use of urine toxicology screens is important in identifying opioid misuse, and doctor-patient discussions about how the responsible use of opioids is defined are essential.

## 9. Opioid Therapy Agreement

Controlled-substance agreements are frequently used in clinics to clarify the roles of the patients and providers and to ultimately improve patient compliance with their opioid medication. These documented agreements provide education and mutual consent among patients and providers and inform patients of their responsibilities when using prescribed pain medication. 

A controlled-substance agreement should state that the patient is only allowed to remain in the program if they adhere to the termed conditions (Appendix D). The following are sample conditions: (1) patients should only use their prescribed medications as directed by their physician; (2) they will be unable to receive replacement medication if lost or stolen; (3) they agree to only receive prescription pain medication from one physician; (4) they will not receive additional medication if their prescription runs out early; (5) they will accept generic brands of prescription medication; (6) if daily function has not improved with their prescription of pain medication, then the physician has the right to taper the patient off the medication; (7) they agree to submit to urine and blood screens to detect use of nonprescribed medications and to verify the presence of prescribed medications; (8) they agree to participate in all aspects of treatment (e.g., physical therapy, psychotherapy, and behavioral medicine); (9) they will only use one pharmacy to fill prescriptions, and agree to count pills from the pharmacy; (10) they will be responsible in maintaining their appointments. Patients should be encouraged to notify their provider if an acute condition necessitates the need to use additional pain medication. 

The core elements to the controlled substances agreement should be made clear so the patient knows exactly what is expected of them. When the patient signs this agreement, they are acknowledging their consent to the proposed treatment plan and agree to adhere to the specific conditions and responsibilities set by the clinic. It is imperative for all individuals placed on chronic opioid therapy to read and sign an opioid therapy agreement. In this way, patients can be informed of their responsibilities and work to remain compliant with their prescribed medication while avoiding complications. Physicians should periodically request that the patients complete an opioid compliance checklist, which serves to remind patients of their responsibilities for using opioids for pain (Appendix E). 

Gourlay and colleagues created a rational approach to opioid therapy for the treatment of chronic pain using universal precautions [82]. This concept was borrowed from infectious disease paradigms. This approach includes a means of identifying and monitoring patients who may be at risk for misusing physician-prescribed medication. In order to properly assess if patients should be considered for long-term opioid therapy, they suggest the following recommended steps: (1) diagnosis with the appropriate differential; (2) psychological assessment, including risk potential for addictive disorder; (3) informed consent and treatment agreement; (4) pain and function assessment; (5) opioid therapy trial; (6) reassessment of pain, function, and behavior (e.g., analgesia, activities of daily living, adverse events); (7) periodic urine screens; (8) review of diagnosis and comorbidities; (9) detailed documentation. A comprehensive pain management center may provide additional assessment and input for patients who were referred by their physicians. Some pain management specialists offer a trilateral agreement with the patient's primary care physician. Once it is determined that the patient has been compliant and stable with their opioids, they can be referred back to their primary care physician for management of their pain. The pain specialist would provide a periodic reevaluation of the patients if necessary.

## 10. Abuse-Deterrent Opioids

Several new opioid formulations that are designed to prevent or deter the abuse of opioids are currently in development, and two have been approved for marketing (morphine sulfate coformulated with naltrexone hydrochloride (Embeda) and a new formulation of the extended-release oxycodone (OxyContin)) (Appendix F). Abuse-deterrent formulations are those that do not necessarily resist tampering but contain substances that are designed to make the formulation less attractive to abusers. Examples of these formulations are Suboxone (buprenorphine coformulated with the opioid antagonist naloxone), Embeda (an extended-release morphine coformulated with the opioid antagonist naltrexone), ELI-216 (an extended-release oxycodone coformulated with naltrexone), and Acurox (an immediate-release oxycodone coformulated with an aversive agent (niacin)). Tamper-resistant formulations are not co-formulated with an antagonist or aversive agent but are designed to be very difficult to crush or dissolve and thus would prevent chewing, snorting, or injecting the medication. Examples include Remoxy, COL-003, TQ-1017, and the reformulation of OxyContin, all of which are extended-release formulations of oxycodone. 

There are still many issues that are yet to be addressed about their integration into clinical practice and their true abuse liability in real-world scenarios. The cost of these new preparations will likely present a challenge, particularly for insurance carriers and pharmacy managers attempting to justify their use based on cost-benefit analyses. The only substantial real-world experience with these types of formulations is with Suboxone even though this combination of buprenorphine and naltrexone is not approved for the treatment of pain. One study suggested dose escalation is typically greater with pure formula opioids and is not associated with the clinical severity of low back pain, or pain resulting from surgery [83]. 

Another study suggested that introduction of Suboxone did not reduce buprenorphine injection in Malaysian substance abusers [84]. Recent findings suggest that Suboxone, which is used by 170,000 people in the US on a daily basis, is still occasionally crushed and injected by abusers (http://www.choosehelp.com/news/getting-high-on-suboxone-the-fda-says-its-happening-ex-nida-director-blames-doctors.html, Accessed April 23, 2010). Yet even with a certain level of abuse, Suboxone therapy is still considered the safest treatment for opioid addiction. One double-blind study compared buprenorphine transdermal system to placebo and found this treatment to be effective for the management of moderate to severe chronic low back pain for patients who have previously received opioids for their pain [85]. The Suboxone example suggests that abuse-deterrent or tamper-resistant formulations are not likely to completely prevent or deter abuse but that the reductions in abuse they provide may be an important incremental step towards safer treatments and safer communities.

## 11. Interventions for High-Risk Patients

Chronic pain patients who show aberrant drug-related behavior often are discontinued from treatment when they are noncompliant with their use of opioids for pain. A randomized trial of patients prescribed opioids for noncancer back pain who showed risk potential for or demonstration of opioid misuse was conducted to see if close monitoring and cognitive behavioral substance misuse counseling could increase overall compliance with opioids [86]. Forty two patients meeting criteria for high risk for opioid misuse were randomized to either standard control (High-Risk Control; *N* = 21) or experimental compliance treatment consisting of monthly urine screens, compliance checklists, and individual and group motivational counseling (High-Risk Experimental; *N* = 21). Twenty patients who met criteria indicating low potential for misuse were recruited to a low-risk control group (Low-Risk Control). Patients were followed for six months and completed pre- and poststudy questionnaires and monthly electronic diaries. Outcomes consisted of the percent with a positive Drug Misuse Index (DMI), which was a composite score of self-reported drug misuse (Prescription Drug Use Questionnaire), physician-reported abuse behavior (Addiction Behavior Checklist), and abnormal urine toxicology results. After six months, significant differences were found between groups with 73.7% of the High-Risk Control patients demonstrating positive scores on the DMI compared with 26.3% from the High-Risk Experimental group and 25.0% from the Low-Risk Controls (*P* < 0.05). The results of this study demonstrate support for the benefits of a brief behavioral intervention in the management of opioid compliance among chronic back pain patients at high-risk for prescription opioid misuse. At followup, none of the subjects was dismissed from the clinic due to aberrant drug behavior, which was possibly a partial effect of the attention from being in a study and completing monthly electronic diaries. Overall, this study demonstrated a positive effect of improving opioid compliance, particularly among those patients at high risk for misuse of opioids. The results of this study are encouraging and suggest that compliance training and very careful monitoring of those patients determined to be at high risk for opioid misuse can be incorporated as part of an anesthesia-based multidisciplinary pain program to help improve compliance with opioids and to reduce the number of those individuals who are discharged from treatment because of aberrant drug-related behavior. Although further research is needed, this trial demonstrates that substantial improvement in compliance with prescription opioids for many high-risk pain patients is possible within a pain management center.

## 12. Future Considerations

As the world population ages and life expectancy increases, greater attention will be given to managing medical comorbidities, including chronic back pain. The practice of pain management will likely be changing within the next five to ten years in a number of ways. First, opioids will continue to be prescribed as a treatment for noncancer back pain; however, a balance will likely be reached in effectively managing pain while also addressing the problems with addiction, overdose, and death that these medications can cause. In line with this change, new formulas of abuse-deterrent opioids will continue to be developed and greater attention will be given to educating physicians and patients about proper dispensing, storing, and disposing practices associated with the use of opioids. Also, healthcare practitioners will be encouraged to have specialty training and certification in the proper dispensing of opioids. Second, software programs with interactive, dynamic education for prescribers, pharmacists, and patients will continue to be developed and made available to the general public. Greater attention will also be given to outcome studies on the risk factors that predict misuse of opioids. 

Third, many other fruitful and exciting areas of study will improve the way pain is managed. Genetics testing holds much promise for the identification of markers for potential opioid misuse. Our understanding of opioid-induced hyperalgesia and craving within certain individuals will also contribute to the identification of sensitive markers for opioid misuse. In particular, complex endogenous chemical reactive systems that affect tolerance and potential opioid misuse will likely be reliably identified. Also, longitudinal investigations on demographic factors such as gender, ethnic origin, and personality predispositions that influence the use of opioids will improve practice guidelines. Fourth, additional studies on other treatments for back pain such as the use of cannabinoids and their role in symptom reduction could add to the current pain management armamentarium. Also, the development of other delivery systems such as topical preparations will offer a wider array of treatment options. The exciting area of nanotechnology to deliver a drug to a particular area should also greatly enhance treatment options. Finally, a greater understanding of how an acute back pain problem develops into a chronic pain syndrome with subtle effects that this process has on centralized mechanisms will open up interventions that are currently not available. Although the management of chronic back pain will always be a challenge, the future will likely hold answers for improved treatment for those who suffer with chronic pain.

## 13. Conclusions

Comprehensive assessment and monitoring is recommended for all patients who are on long-term opioid therapy for chronic back pain. The close monitoring of patients who are at greatest risk for misuse of their prescribed medication should contain a treatment protocol that includes an opioid agreement, regular urine toxicology screens, compliance checklists, pill counts, and, if indicated, motivational counseling. Careful monitoring and use of abuse-deterrent opioids will hopefully decrease the abuse potential of prescribed opioids; however, risk of opioid misuse and addiction will remain, and close attention to screening and documentation of treatment outcomes will continue to be the gold standard of opioid therapy.

## Appendices

### A. Terminology Definitions Related to Substance Use Disorders

Substance Misuse The use of any drug in a manner other than how it is indicated or prescribed.

Substance Abuse The use of any substance when such use is unlawful, or when such use is detrimental to the user or others.

Addiction A primary, chronic, neurobiologic disease that is characterized by behaviors that include one of more of the following: impaired control over drug use, compulsive use, continued use despite harm, and craving. Addiction is generally understood to be a chronic condition from which recovery is possible; however, the underlying neurobiologic dysfunction, once manifested, is believed to persist.

Physical Dependence A state of adaptation that is manifested by a drug class-specific withdrawal syndrome that can be produced by abrupt cessation, rapid dose reduction, or decreasing blood levels of the drug and/or by administration of an antagonist.

Tolerance A state of adaptation in which exposure to a drug induces changes that result in diminution of one or more of the drug's effects over time.

Aberrant Drug-Related Behavior Behavior suggestive of a substance abuse and/or addiction disorder. Examples are selling prescription drugs, prescription forgery, stealing or “borrowing” drugs from others, injecting oral formulations, obtaining prescription drugs from nonmedical sources, multiple episodes of prescription “loss,” repeatedly seeking prescriptions from other clinicians, evidence of deterioration in function (work, home, family), and repeated resistance to change therapy despite evidence of physical and psychological problems.

Pseudoaddiction An iatrogenic misinterpretation caused by undertreated pain that is identified by the clinician as inappropriate drug seeking behavior. Misuse behavior ceases when adequate pain relief is provided. This is not a diagnosis but rather a description of a clinical interaction.

### B. List of Opioid Risk Screening Tools

- Screener and Opioid Assessment for Patients in Pain-Revised (SOAPP-R).
- Current Opioid Misuse Measure (COMM).
- Opioid Risk Tool (ORT).
- Diagnosis, Intractability, Risk, and Efficacy (DIRE).
- Screening Instrument for Substance Abuse Potential (SISAP).
- The Pain Assessment and Documentation Tool (PADT).

### C. Risk Factors for Opioid Misuse

- Family history of substance abuse.
- Personal history of substance abuse.
- Young age.
- History of criminal activity and/or legal problems including DUIs.
- Regular contact with high-risk people or high-risk environments.
- Problems with past employers, family members, and friends (mental disorder).
- Risk taking or thrill seeking behavior.
- Heavy tobacco use.
- History of severe depression or anxiety.
- Psychosocial stressors.
- Prior drug and/or alcohol rehabilitation.

### D. Example of a Controlled Substance Agreement

This agreement relates to my use of controlled substances for chronic pain prescribed by a physician. I have been informed and understand the policies regarding the use of controlled substances. I understand that I will be provided controlled substances while actively participating in this program only if I adhere to the following conditions:

1. I will use the substances only as directed by my physician.

(2) I will not expect to receive replacement medications for any medications that I have lost or have been stolen. A police report must be produced for any consideration of replacement of any lost or stolen medication.

(3) I will receive controlled substances only from one physician. Information that I have obtained controlled substances from another physician without prior knowledge will lead to discontinuation of treatment.

(4) I will not expect to receive additional medication prior to the time of my next scheduled refill, even if my prescription runs out.

(5) I will accept generic brands of my prescription medication, where determined appropriate by my physician.

(6) If it appears to the physician that there are no demonstrable benefits to my daily function or quality of life from the controlled substance, I will gradually taper my medication as directed by my prescribing physician.

(7) I agree to submit to urine and blood screens to detect the use of nonprescribed controlled medications (including “street” drugs) and verify the presence of my prescribed medications at any time.

(8) I recognize that my chronic pain represents a complex problem, which may benefit from physical therapy, psychotherapy, and behavioral medicine strategies. I also recognize that my active participation in the management of my pain is extremely important. I agree to actively participate in all aspects of my treatment to maximize functioning and improve coping with my condition.

(9) I agree to schedule and keep scheduled follow-up appointments with my physician at recommended intervals. I understand that failure to keep appointments may lead to discontinuation of treatment.

(10) I am responsible for keeping track of the amount of medication that I have left and to plan ahead for arranging the refill of my prescriptions in a timely manner so I will not run out of medications.

(11) I agree to use one pharmacy for filling all my prescriptions except in case of emergency.

(12) I will agree to count my pills that I receive from pharmacy and will ensure that the correct amount is received. I understand that I will not expect my physician to cover me for any shortage of medication. Any shortage found must be discussed immediately upon my receiving the prescription with the pharmacist.

(13) If I violate any of the above conditions, my obtaining prescriptions and/or treatment may be terminated.

(14) If I violate any of the above conditions and the violation involves obtaining controlled substances, or any prescription, for my pain condition from another individual, or, if I engage in any illegal activity such as altering a prescription, I understand that the incident may be reported by my physician. As deemed appropriate for the violation, my physician may report my violations to other physicians caring for me, local medical facilities, pharmacies, local police departments, and/or Drug Enforcement Agencies.

(15) I can designate up to two other people to pick up my prescriptions. I understand that I must notify my physician in advance each and every time a prescription is refilled if an alternate person will be picking up the prescription. Failure to do this could result in the prescription not being released. The names of these people must be entered at the bottom of this contract.

(16) This Controlled Substances Contract will become part of my permanent medical record.

This agreement will supersede all other agreements.

By signing below, I indicate that I understand and agree to all the terms of the above agreement. I have received a copy of this for my own records.

** Patient: _______________________________**
** Date and Time: _______________________________**
** Physician:_______________________________**
** Date and Time: ______________________________**

Names of the people that I designated to pick up my prescriptions
  #1 **____________________________________ ** 
  #2 **____________________________________ **

### E. Opioid Compliance Checklist

Print name: **_______________________________**
Date: **_______________________________**

Please answer the following questions as honestly as possible:

Over the past month have you:

1. Taken your opioid medication other than the way they were prescribed?  Yes No
2. Used more than one pharmacy to fill your opioid prescriptions?  Yes No
3. Received opioid prescriptions from more than one provider?  Yes No
4. Lost or misplaced your opioid medication?  Yes No
5. Run out of your pain medication early?  Yes No
6. Missed any scheduled medical appointments?  Yes No
7. Borrowed opioid medication from others?  Yes No
8. Used any illegal or unauthorized substances? Yes No
9. Taken the highest possible degree of care of your prescription medication?  Yes No
10. Taken any unauthorized substance that might be found in your urine?  Yes No
11. Been involved in any activity that may be dangerous to you or someone else if you felt drowsy or were not clear thinking?  Yes No
12. Been completely honest about your personal drug use?  Yes No

Please explain anything further below. Thank you.

Signed: _____________
Date: _____________

### F. Abuse-Deterrent Opioids

- Current name: Remoxy.
- Active Drug: Oxycodone (extended-release).
- Type of Formulation: Gelatin capsule containing highly viscous liquid.
- Manufacturer: Pain Therapeutics; Pfizer (formerly King Pharmaceutical).
- Current name: COL-003.
- Active Drug: Oxycodone (extended-release).
- Type of Formulation: Multiparticulate matrix with particles in waxy excipient base.
- Manufacturer: Collegium Pharmaceutical.
- Current name: ELI-216.
- Active Drug: Oxycodone (extended-release).
- Type of Formulation: Capsule containing separate oxycodone and naltrexone pellets.
- Manufacturer: Elite Pharmaceuticals.
- Current name: Unknown (reformulation of OxyContin).
- Active Drug: Oxycodone (extended-release).
- Type of Formulation: Hard polymer that transforms into a viscous gel with hydration.
- Manufacturer: Purdue Pharma.
- Current name: Embeda (ALO-01).
- Active Drug: Morphine (extended-release)
- Type of Formulation: Pellets of morphine surrounding an inner core of naltrexone.
- Manufacturer: Pfizer (formerly King Pharmaceutical).
- Current name: TQ-1017.
- Active Drug: Tramadol (extended-release).
- Type of Formulation: Transforms into viscous substance in the presence of solvents.
- Manufacturer: TheraQuest Biosciences.
- Current name: Acurox.
- Active Drug: Oxycodone (immediate-release).
- Type of Formulation: Coformulated with subtherapeutic doses of niacin.
- Manufacturer: Acura Pharmaceuticals, Pfizer (former King Pharmaceutical).
